# Whole brain irradiation–induced endothelial dysfunction in the mouse brain

**DOI:** 10.1007/s11357-023-00990-4

**Published:** 2023-11-13

**Authors:** Tamas Kiss, Anna Ungvari, Rafal Gulej, Ádám Nyúl-Tóth, Stefano Tarantini, Zoltan Benyo, Boglarka Csik, Andriy Yabluchanskiy, Peter Mukli, Anna Csiszar, Zoltan Ungvari

**Affiliations:** 1 Vascular Cognitive Impairment, Neurodegeneration and Healthy Brain Aging Program, Department of Neurosurgery, University of Oklahoma Health Sciences Center, Oklahoma City, OK USA; 2 Oklahoma Center for Geroscience and Healthy Brain Aging, University of Oklahoma Health Sciences Center, Oklahoma City, OK USA; 3 International Training Program in Geroscience, Doctoral School of Basic and Translational Medicine/Department of Public Health, Semmelweis University, Budapest, Hungary; 4 First Department of Pediatrics, Semmelweis University, Budapest, Hungary; 5 International Training Program in Geroscience, Doctoral School of Basic and Translational Medicine/Department of Translational Medicine, Semmelweis University, Budapest, Hungary; 6 Eötvös Loránd Research Network and Semmelweis University (ELKH-SE) Cerebrovascular and Neurocognitive Disorders Research Group, Budapest, Hungary; 7 Department of Public Health, Semmelweis University, Semmelweis University, Budapest, Hungary; 8 Stephenson Cancer Center, University of Oklahoma, Oklahoma City, OK USA; 9 Department of Health Promotion Sciences, College of Public Health, University of Oklahoma Health Sciences Center, Oklahoma City, OK USA

**Keywords:** Senescence, WBI, WBRT, whole brain radiation therapy, aging, vascular cognitive impairment, VCI, dementia

## Abstract

Whole brain irradiation (WBI), also known as whole brain radiation therapy (WBRT), is a well-established treatment for multiple brain metastases and as a preventive measure to reduce the risk of recurrence after surgical removal of a cerebral metastasis. However, WBI has been found to lead to a gradual decline in neurocognitive function in approximately 50% of patients who survive the treatment, significantly impacting their overall quality of life. Recent preclinical investigations have shed light on the underlying mechanisms of this adverse effect, revealing a complex cerebrovascular injury that involves the induction of cellular senescence in various components of the neurovascular unit, including endothelial cells. The emergence of cellular senescence following WBI has been implicated in the disruption of the blood-brain barrier and impairment of neurovascular coupling responses following irradiation. Building upon these findings, the present study aims to test the hypothesis that WBI-induced endothelial injury promotes endothelial dysfunction, which mimics the aging phenotype. To investigate this hypothesis, we employed a clinically relevant fractionated WBI protocol (5 Gy twice weekly for 4 weeks) on young mice. Both the WBI-treated and control mice were fitted with a cranial window, enabling the assessment of microvascular endothelial function. In order to evaluate the endothelium-dependent, NO-mediated cerebral blood flow (CBF) responses, we topically administered acetylcholine and ATP, and measured the resulting changes using laser Doppler flowmetry. We found that the increases in regional CBF induced by acetylcholine and ATP were significantly diminished in mice subjected to WBI. These findings provide additional preclinical evidence supporting the notion that WBI induces dysfunction in cerebrovascular endothelial cells, which in turn likely contributes to the detrimental long-term effects of the treatment. This endothelial dysfunction resembles an accelerated aging phenotype in the cerebrovascular system and is likely causally linked to the development of cognitive impairment. By integrating these findings with our previous results, we have deepened our understanding of the lasting consequences of WBI. Moreover, our study underscores the critical role of cerebromicrovascular health in safeguarding cognitive function over the long term. This enhanced understanding highlights the importance of prioritizing cerebromicrovascular health in the context of preserving cognitive abilities.

## Introduction

Brain metastases rank as the second most frequent occurrence in cancer patients, with an incidence ranging from 10 to 30% among individuals with systemic malignancies [1, 2]. Whole brain irradiation (WBI) represents a widely employed therapeutic approach for patients afflicted with multiple brain metastases. While WBI effectively prolongs overall survival in individuals with cancer, it is distressing to note that more than half of the long-term survivors experience a gradual deterioration in cognitive function following WBI [3–9]. This cognitive decline significantly diminishes their quality of life and contributes to escalated healthcare expenses. Intriguingly, laboratory studies on animals have corroborated these clinical findings, as they demonstrate a progressive impairment of cognitive performance in response to WBI, thereby simulating the cognitive side effects observed in patients subjected to WBI treatment [3, 8, 10–14].

Recent advancements in preclinical investigations have provided valuable insights into the underlying mechanisms behind these deleterious effects, unveiling a complex injury within the cerebrovascular system [14–17]. This injury entails the activation of cellular senescence in diverse constituents of the neurovascular unit, including endothelial cells [15]. The emergence of cellular senescence subsequent to WBI has been linked to disruptions in the blood-brain barrier and impairments in neurovascular coupling responses post-irradiation [15].

It is worth noting that post-mitotic cells, such as mature neurons, are generally regarded as radioresistant. In contrast, proliferating cells within the neurovascular unit, specifically microvascular endothelial cells, demonstrate sensitivity to the damaging effects of ionizing radiation [15, 18]. Normal endothelial function in the cerebral microcirculation plays a multifaceted role in the preservation of cognitive health. An accumulating body of preclinical and clinical evidence highlights the critical, complex role of radiation-induced neurovascular and cerebromicrovascular injury in the manifestation of cognitive decline subsequent to WBI [13, 19–22]. Importantly, WBI-induced neurovascular injury associates with impaired neurovascular coupling responses [14, 15] and a decline in capillary density within the hippocampus, a phenomenon referred to as “cerebromicrovascular rarefaction” [19–21]. Maintaining microvascular health is also crucial for preserving the integrity of the blood-brain barrier (BBB) [23, 24]. Strong evidence suggests that WBI and γ-irradiation-induced microvascular injury contribute to BBB disruption [25–33], leading to neuroinflammation [34, 35].

One key aspect of endothelial function is the crucial role of endothelial NO-mediated vasodilation in regulating and maintaining cerebral blood flow (CBF). Endothelial cells release NO in response to various stimuli, including neurotransmitters and gliotransmitters (e.g., acetylcholine, ATP) and shear stress. NO acts as a potent vasodilator, relaxing the vascular smooth muscle cells and pericytes surrounding cerebral blood vessels, thereby decreasing resistance and increasing blood flow to active brain regions. This dynamic regulation of CBF is essential for supplying oxygen and nutrients to the brain and for effective wash-out of toxic metabolic by-products, ensuring optimal neuronal activity and synaptic plasticity. Furthermore, endothelial NO-mediated vasodilation is integral to the preservation of neurovascular coupling response, a critical hoeostatic process that ensures that increases in neuronal activity are matched with enhanced CBF to sustain neuronal function [36–51]. Importantly, intact endothelial function and NO-mediated vasodilation serve as a protective mechanism against ischemic events in the brain. The ability of endothelial cells to respond and dilate blood vessels helps maintain optimal blood flow even under conditions of reduced oxygen supply. Inadequate endothelial function compromises this protective response, rendering the brain more susceptible to ischemic damage and impairments in cognitive function. Taken together, normal endothelial function in the cerebral microcirculation is critical for the preservation of cognitive health. Despite the importance of endothelial NO-mediated vasodilation in regulation and maintenance of adequate CBF, the effects of WBI on it have not been well characterized.

Building upon the aforementioned findings, the present study aims to test the hypothesis that WBI-induced endothelial injury promotes endothelial dysfunction, which mimics the aging phenotype. To investigate this hypothesis, we employed a clinically relevant fractionated WBI protocol (5 Gy twice weekly for 4 weeks) on young mice. Both the WBI-treated and control mice were fitted with a cranial window, enabling the assessment of microvascular endothelial function. In order to evaluate the endothelium-dependent, NO-mediated cerebral blood flow (CBF) responses, we topically administered acetylcholine and ATP, and measured the resulting changes using laser Doppler flowmetry.

## Materials and methods

### Experimental animals and experimental design

C57BL/6J (3 months old, *n* = 20) male mice were purchased from the Jackson Laboratories (Bar Harbor, ME) and housed three per cage in the specific pathogen free animal facility at the University of Oklahoma Health Sciences Center (OUHSC). Animals were kept on a 12-h light/dark cycle and fed standard rodent chow and water ad libitum, following standard husbandry techniques. One week before radiation treatment, mice were transferred to the conventional facility (OUHSC) and housed under similar conditions. Mice were anesthetized and subjected to clinical series of WBI (*n* = 8, 5 Gy twice weekly for a total cumulative dose of 40 Gy) or used as a control group (*n* = 8). Mice were left to recover for 3 months in the original environment. At the end of the recovery period, mice were experimentally tested for cerebromicrovascular endothelial function. All animal protocols were approved by the Institutional Animal Care and Use Committee of OUHSC.

### Fractionated whole brain irradiation protocol

After acclimating to the conventional facility for 1 week, mice were randomly assigned to either control or irradiated groups. Animals were weighed and anesthetized via i.p. injection of ketamine and xylazine (100/15 mg per kg). Mice in the irradiated group were subjected to clinically relevant WBI (5 Gy twice weekly for a total cumulative dose of 40 Gy) [17]. Radiation was administered using a ^137^Cesium gamma irradiator (GammaCell 40, Nordion International). A Cerrobend® shield was utilized to minimize exposure outside the brain. The radiation dose received by the mice was verified using film dosimetry, as described [13, 14, 16, 21].

### Assessment of cerebromicrovascular endothelial function

To determine how accelerated brain senescence induced by WBI affects cerebromicrovascular function, CBF responses elicited by the endothelium-dependent vasodilator agents acetylcholine and ATP were assessed at 3 months post WBI treatment. Mice in both groups were anesthetized with isoflurane (4% induction and 1% maintenance), endotracheally intubated, and ventilated (MousVent G500; Kent Scientific Co, Torrington, CT). A thermostatic heating pad (Kent Scientific Co, Torrington, CT) was used to maintain rectal temperature at 37 °C [52]. The right femoral artery was canulated for arterial blood pressure measurement (Living Systems Instrumentations, Burlington, VT). The blood pressure was within the physiological range throughout the experiments (90–110 mmHg). Mice were immobilized and placed on a stereotaxic frame (Leica Microsystems, Buffalo Grove, IL), and the scalp and periosteum were pulled aside. Mice were equipped with an open cranial window, and changes in CBF were assessed above the left somatosensory cortex using a laser Doppler probe (Transonic Systems Inc., Ithaca, NY), as described [39, 52, 53]. The cranial window was superfused with artificial cerebrospinal fluid (ACSF, composition: NaCl 119 mM, NaHCO_3_ 26.2 mM, KCl 2.5 mM, NaH_2_PO_4_ 1 mM, MgCl_2_ 1.3 mM, glucose 10 mM, CaCl_2_ 2.5 mM, pH = 7.3, 37 °C). To assess microvascular endothelial function, endothelium-dependent, NO mediated CBF responses to topical administration of acetylcholine (ACh; 10^−5^ mol/L)), and ATP (10^−6^ mol/L)) were obtained following established protocols [45]. In each study, the experimenter was blinded to the treatment of the animals. At the end of the experiments, the animals were killed by decapitation. All reagents used in this study were purchased from Sigma-Aldrich (St Louis, MO) unless otherwise indicated.

### Statistical analysis

The data are presented as means ± standard error of the mean (SEM). Statistical analysis was conducted using the GraphPad Prism 8 software (La Jolla, CA, USA) [50, 54]. Student’s two-sample *T*-test was employed to compare the experimental results, and differences were deemed significant at *p* < 0.05.

## Results

### WBI induces persisting endothelial dysfunction

**Fig. 1:**
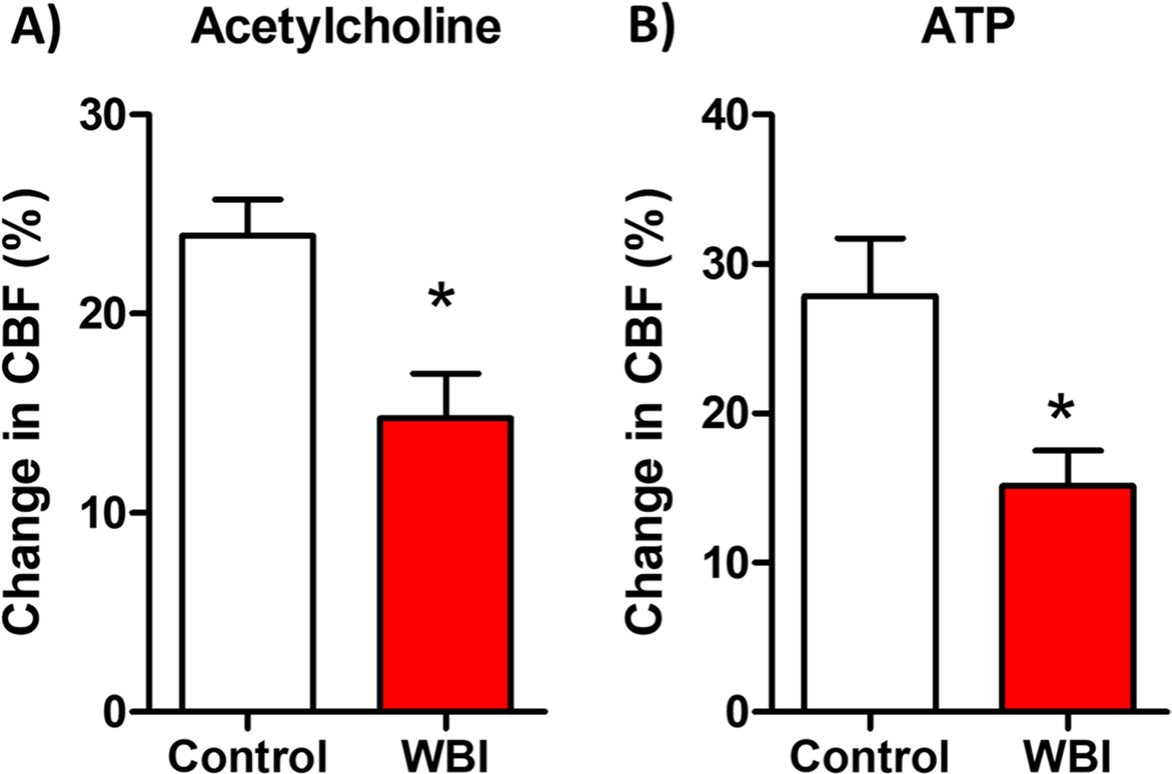
Whole brain irradiation (WBI) induces persistent endothelial dysfunction in the cerebral circulation. Laser Doppler probe measurements were utilized to assess changes in cerebral blood flow (CBF) in the cerebral cortex of mice following topical perfusion of the endothelium-dependent vasodilator agents acetylcholine (**A**) and ATP (**B**) at 3 months post-WBI. Bar graphs depict summary data. Importantly, both acetylcholine and ATP-induced CBF responses in the somatosensory cortex were significantly reduced in WBI-treated mice compared to control animals, indicative of impaired endothelial function persisting at 3 months post-irradiation. Data presented are mean ± S.E.M. (*n* = 8 for each data point). *P < 0.05 vs. control

In order to determine whether WBI results in persisting cerebromicrovascular endothelial dysfunction, we assessed endothelium-dependent vasodilation in the cerebral cortex in mice at 3 months post-WBI. We found that CBF responses in the somatosensory cortex elicited by acetylcholine were significantly decreased in WBI-treated mice compared to control animals indicating impaired endothelial function at 3 months post-irradiation (Fig. 1A). In control mice, topical administration of ATP also resulted in significant CBF increases in the somatosensory cortex, whereas these responses were significantly attenuated in WBI-treated mice (Fig. 1B).

## Discussion

Our study aimed to investigate the hypothesis that WBI-induced endothelial injury promotes endothelial dysfunction, mimicking the aging phenotype. The results of our study provide compelling evidence of persisting cerebromicrovascular endothelial dysfunction following WBI. At 3 months post-irradiation, we observed a significant decrease in endothelium-dependent vasodilation in the cerebral cortex of WBI-treated mice compared to control animals. Specifically, the CBF responses elicited by acetylcholine were markedly impaired in WBI-treated mice, indicating compromised endothelial function. Additionally, the CBF responses induced by ATP, another endothelium-dependent vasodilator, were significantly attenuated in WBI-treated mice, further confirming the presence of endothelial dysfunction.

**Fig. 2:**
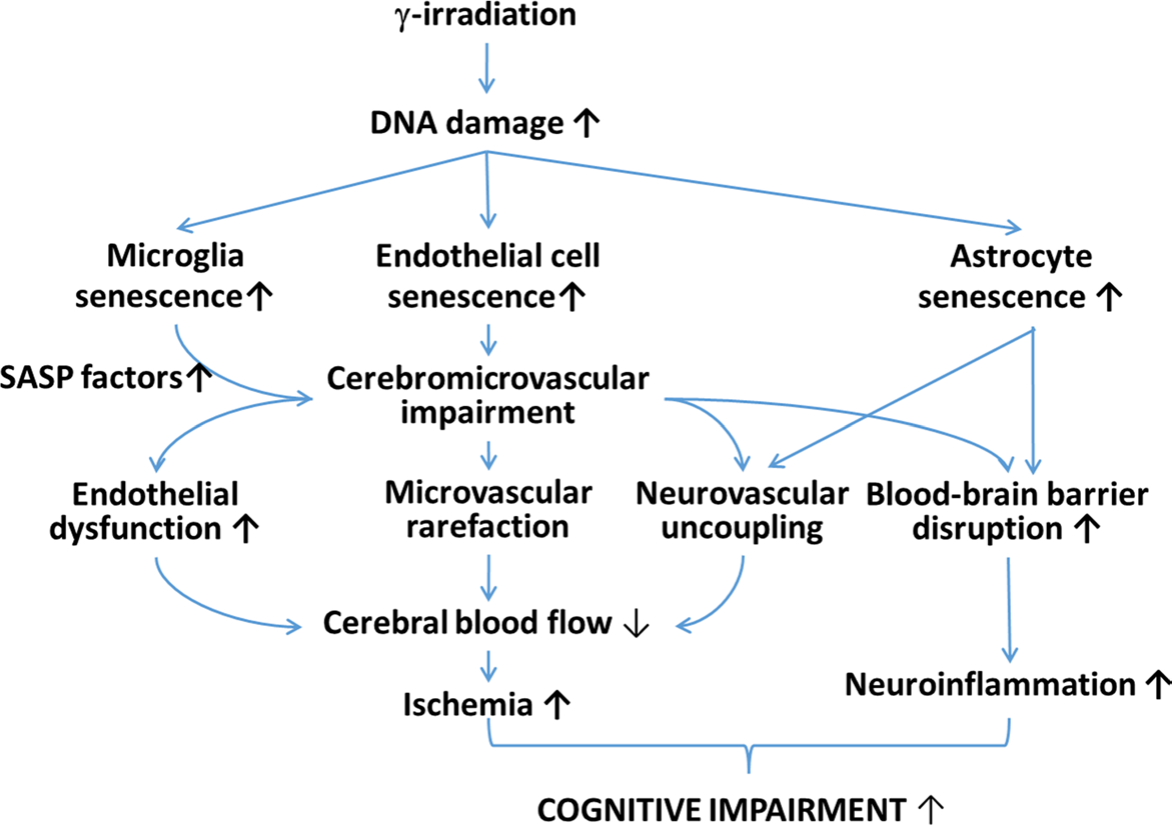
Proposed scheme for the contribution of neurovascular senescence and endothelial dysfunction to WBI-induced cognitive decline. γ-irradiation induced DNA damage and cellular senescence in cells of the neurovascular unit, including endothelial cells, perivascular microglia, and astrocytes. Endothelial senescence results in functional and structural impairment of the cerebral microcirculation, including endothelial dysfunction, impairment of neurovascular coupling responses, and microvascular rarefaction, all of which contribute to a significant decline in cerebral blood flow (CBF). γ-irradiation-induced neurovascular senescence disrupts the blood brain barrier, exacerbating neuroinflammation. Heightened inflammatory status of the neurovascular unit, due to endothelial senescence and the increased secretion of pro-inflammatory SASP factors from senescent microglia and astrocytes exacerbates cerebromicrovascular dysfunction and neuroinflammation. The resulting ischemic and inflammatory foci play a role in the pathogenesis of cognitive impairment. The model predicts that the aforementioned senescence-related structural and functional cerebromicrovascular alterations synergize to promote cognitive impairment in patients treated with WBI

These findings align with previous studies demonstrating the detrimental effects of WBI on cerebromicrovascular health (Fig. 2). Endothelial cells play a crucial role in regulating CBF flow through the release of NO, a potent vasodilator, which relaxes vascular smooth muscle cells and pericytes. Impaired endothelial NO-mediated vasodilation decreases basal CBF and compromises the dynamic regulation of regional CBF, impeding the delivery of oxygen, nutrients to active brain regions, and the effective clearance of metabolic by-products. Consequently, this disruption in CBF can adversely affect neuronal activity and synaptic plasticity, contributing to cognitive impairment [41].

The observed persisting endothelial dysfunction in WBI-treated mice adds to the growing body of evidence implicating cerebromicrovascular and neurovascular injury in the development of cognitive decline following WBI (Fig. 2). Our findings extend the results of previous studies demonstrating a decline in capillary density within the hippocampus, the cerebral cortex, and/or the white matter in response to WBI [13, 17, 19–21, 55]. Irradiation-induced microvascular rarefaction likely exacerbates the negative effects of impaired endothelium-mediated microvascular dilation and neurovascular coupling responses, compromising cerebral blood supply and, subsequently, cognition (Fig. 2). Furthermore, intact endothelial function is crucial for maintaining the integrity of the blood-brain barrier (BBB). Disruption of the BBB, as observed in WBI and γ-irradiation-induced microvascular injury [25–33], can lead to neuroinflammation and contribute to the genesis of cognitive impairment [34, 35] (Fig. 2).

One crucial mechanism underlying the detrimental effects of γ-irradiation on endothelial cells involves the generation of persistent DNA damage, triggering a chronic stress response known as cellular senescence [15, 18, 56, 57] (Fig. 2). Compelling evidence indicates that cerebromicrovascular endothelial cells (CMVECs) are particularly vulnerable to DNA damage induced by γ-irradiation, leading to the acquisition of senescent phenotypes in cultured CMVECs [18]. When cellular senescence is induced in culture, irradiated CMVECs undergo cell cycle arrest, display significant morphological changes, and develop a senescence-associated secretory phenotype (SASP), characterized by an increased secretion of pro-inflammatory cytokines [18]. The growing preclinical evidence highlights the contribution of senescent endothelial cells and their SASPs to the pathogenesis of microvascular disorders and cognitive decline associated with aging [49]. Moreover, studies in genetically modified mice, where cells expressing the senescence marker p16INK4A were depleted, have shown promising outcomes. These mice exhibit prolonged median lifespan, improved overall health, restored microvascular function, and enhanced cognition [58–67], providing further support for the pivotal role of cellular senescence in brain and cerebromicrovascular aging [49, 68–70].

Building upon these findings, our recent studies have demonstrated that eliminating senescent cells in WBI-treated mice yields protective effects on the regulation of cerebral blood flow and preserves cognitive performance [15]. These observations highlight the significance of targeting cellular senescence as a potential therapeutic strategy to mitigate the adverse consequences of WBI on cerebromicrovascular health and cognitive function (Fig. 2). Expanding upon these significant findings, our recent studies have provided compelling evidence that the elimination of senescent cells in WBI-treated mice confers remarkable protective effects on multiple aspects of cerebromicrovascular health and cognitive function [15]. Notably, the elimination of senescent cells was found to rescue neurovascular coupling responses [15]. The impaired neurovascular coupling observed in WBI-treated mice was ameliorated when senescent cells were targeted, suggesting a restoration of the intricate interplay between neurons, astrocytes, and endothelial cells within the neurovascular unit. This restoration of neurovascular coupling responses is of paramount importance as it ensures an adequate supply of oxygen and nutrients to active brain regions, thereby supporting optimal neuronal function. Furthermore, the elimination of senescent cells also exhibited a profound impact on preserving the integrity of the blood-brain barrier (BBB) in WBI-treated mice (Gulej et al. 2023 *in press*). Targeting senescent cells not only improves vasodilator function of the endothelial cells and restores BBB integrity but also likely mitigates the associated neuroinflammatory responses, thus providing a holistic therapeutic avenue to alleviate the genesis of cognitive decline following WBI treatment. These observations hold immense potential for the development of novel translational therapeutic strategies that can counteract the adverse consequences of WBI on cerebromicrovascular function and cognitive performance, ultimately improving the quality of life for individuals undergoing brain irradiation treatments. In the cerebral microcirculation, CMVECs are interconnected via specialized channels called gap junctions, facilitating the transfer of solutes and cytoplasmic signals [71]. This arrangement enables the formation of a functional syncytium [71], where a single senescent endothelial cell can directly influence the function and characteristics of neighboring CMVECs. Moreover, senescence can propagate within the microcirculation through the exposure of neighboring cells to paracrine factors released by senescent CMVECs, known as the senescence-associated secretory phenotype (SASP). This phenomenon, referred to as paracrine senescence or senescence-induced senescence, can result in the spread of senescence to adjacent cells. As a consequence, the increased presence of senescent endothelial and astroglial cells in the brains of WBI-treated mice is anticipated to impact a substantial portion of the cerebral microcirculatory network. Conversely, the elimination of senescent cells through treatment with senolytics is likely to confer protective effects throughout the entire cerebral microcirculation.

In this study, we have endeavored to provide valuable insights into the effects of WBI on cerebromicrovascular endothelial function. However, it is imperative to acknowledge certain limitations that impact the interpretation and generalizability of our findings, including the lack of comprehensive data on systemic cardiovascular function. Future research could also explore WBI-induced transcriptomic changes in endothelial cells, including changes in mRNA and protein expression of NOS isoforms and potential alterations of the local renin-angiotensin II system [72, 73].

In conclusion, our results support the hypothesis that WBI-induced endothelial injury promotes endothelial dysfunction, resembling the aging phenotype. The observed persisting endothelial dysfunction in WBI-treated mice contributes to our understanding of the long-term consequences of WBI and the role of cerebromicrovascular health in preserving cognitive function. Further research is warranted to elucidate the precise mechanisms underlying WBI-induced, senescence-related endothelial dysfunction, and its impact on cognitive decline, with the ultimate goal of developing targeted therapeutic strategies to mitigate these adverse effects.

## Data Availability

All data generated or analyzed during this study are included in the manuscript and/or in its supplementary information files. The raw datasets used and/or analyzed during the current study are available from the corresponding author on reasonable request.
